# The Depolarization-Evoked, Ca^2+^-Dependent Release of Exosomes From Mouse Cortical Nerve Endings: New Insights Into Synaptic Transmission

**DOI:** 10.3389/fphar.2021.670158

**Published:** 2021-07-22

**Authors:** Guendalina Olivero, Francesca Cisani, Danilo Marimpietri, Daniela Di Paolo, Maria Cristina Gagliani, Marina Podestà, Katia Cortese, Anna Pittaluga

**Affiliations:** ^1^ Department of Pharmacy, DIFAR, Pharmacology and Toxicology Section, University of Genoa, Genoa, Italy; ^2^ Stem Cell Laboratory and Cell Therapy Center, IRCCS Istituto Giannina Gaslini, Genoa, Italy; ^3^ Laboratory of Experimental Therapies in Oncology, IRCCS Istituto Giannina Gaslini, Genoa, Italy; ^4^ Department of Experimental Medicine, DIMES, Human Anatomy Section, University of Genoa, Genoa, Italy; ^5^ Department of Pharmacy, DIFAR, Pharmacology and Toxicology Section, Centre of Excellence for Biomedical Research, 3Rs Center, University of Genoa, Genoa, Italy; ^6^ IRCCS Ospedale Policlinico San Martino, Genoa, Italy

**Keywords:** synaptosomes, exosomes, calcium dependency, glutamate release, GABAB receptor

## Abstract

Whether exosomes can be actively released from presynaptic nerve terminals is a matter of debate. To address the point, mouse cortical synaptosomes were incubated under basal and depolarizing (25 mM KCl-enriched medium) conditions, and extracellular vesicles were isolated from the synaptosomal supernatants to be characterized by dynamic light scattering, transmission electron microscopy, Western blot, and flow cytometry analyses. The structural and biochemical analysis unveiled that supernatants contain vesicles that have the size and the shape of exosomes, which were immunopositive for the exosomal markers TSG101, flotillin-1, CD63, and CD9. The marker content increased upon the exposure of nerve terminals to the high-KCl stimulus, consistent with an active release of the exosomes from the depolarized synaptosomes. High KCl-induced depolarization elicits the Ca^2+^-dependent exocytosis of glutamate. Interestingly, the depolarization-evoked release of exosomes from cortical synaptosomes also occurred in a Ca^2+^-dependent fashion, since the TSG101, CD63, and CD9 contents in the exosomal fraction isolated from supernatants of depolarized synaptosomes were significantly reduced when omitting external Ca^2+^ ions. Differently, (±)-baclofen (10 µM), which significantly reduced the glutamate exocytosis, did not affect the amount of exosomal markers, suggesting that the GABA_B_-mediated mechanism does not control the exosome release. Our findings suggest that the exposure of synaptosomes to a depolarizing stimulus elicits a presynaptic release of exosomes that occurs in a Ca^2+^-dependent fashion. The insensitivity to the presynaptic GABA_B_ receptors, however, leaves open the question on whether the release of exosomes could be a druggable target for new therapeutic intervention for the cure of synaptopathies.

## Introduction

Extracellular vesicles (EVs) have emerged in the last 3 decades as a novel way of cell-to-cell communication (Raposo and Stoorvogel, 2013). Exosomes are nanosized EVs (30–100 nm in diameter) of endosomal origin, secreted by most cells in the body, known to vehiculate complex cargoes, including proteins, lipids, and nucleic acids, whose composition is supposed to depend on the cell they originate from. Once released in the extracellular fluids, they are taken up by the selected target cells, influencing their functions (Mathieu et al., 2019).

Exosomes also mediate intercellular communication in the CNS, where they are actively released by all the CNS cells, including astrocytes (Verkhratsky et al., 2016), microglia (Paolicelli et al., 2018), oligodendrocytes (Frühbeis et al., 2013), and neurons (Chivet et al., 2012). Data in the literature demonstrated that the exosomal trafficking has important implications in CNS physiology and pathology, from supporting neurogenesis, neuroprotection, and brain homeostasis (Smalheiser, 2007; Saeedi et al., 2019; Schiera et al., 2020) to favoring the cell-to-cell spreading of “pathogenic proteins” (*β* amyloid peptides, tau, and prions; Holm et al., 2018; Caruso Bavisotto et al., 2019).

Emerging evidence suggests that exosomes are also involved in the modulation of synaptic transmission and plasticity. Cultured cortical neurons release exosomes, mainly from the somata and the dendrites, and this secretion is increased by depolarization and strictly dependent on synaptic glutamatergic activity. Furthermore, neuron-derived exosomes carry selected proteins involved in synaptic transmission, such as GluA2/3 AMPA receptor subunits, suggesting a new mechanism for regulating synaptic strength after neuronal activation (Faurè et al., 2006; Lachenal et al., 2011). Whether exosomes can also be actively released from presynaptic neuronal terminals of the CNS mammalian synapses, however, has not been clarified so far, although some evidence supports the hypothesis (Xu et al., 2013; Janas et al., 2016; Wang et al., 2017). As a matter of fact, at the *Drosophila* neuromuscular junction, which represents a model of presynaptic structure, a trans-synaptic transferring of the Wnt-family signaling protein Wingless (Wg) was reported to occur through exosomes released from the synaptic boutons and containing the Wg-binding protein Evenness Interrupted (Korkut et al., 2009; Koles et al., 2012). In the same model, synaptotagmin-4 was also found to be delivered through exosomes from the presynaptic motor neuron to the muscle fiber to mediate the activity-dependent synaptic growth (Korkut et al., 2013).

Synaptosomes are isolated nerve endings which carry the structural features and the properties of the *in vivo* neuronal terminals they originate from. They are widely recognized as an *in vitro* model for selectively studying the molecular events occurring presynaptically, particularly the release of neurotransmitters and its modulation by presynaptic receptors (Raiteri, 2001; Raiteri, 2008; Langer, 2008).

Our study aimed at investigating whether synaptosomes can release EVs having the morphological and proteomic features of exosomes. The results support the conclusion, suggesting new unexpected aspects of the synaptic transmission that could represent the targets of new therapeutic interventions to control and restore the efficiency of the central synaptic connections.

## Methods

### Animals

Mice (male, strain C57BL/6J) were obtained from Charles River (Calco, Italy) and housed in the animal facility of the Department of Pharmacy (DIFAR), Pharmacology and Toxicology Section (Genoa, Italy), under controlled environmental conditions (ambient temperature = 22°C, humidity = 40%) on a 12-h light/dark cycle with food and water *ad libitum*. The animal care and experimental procedures complied with the European Communities Parliament and Council Directive of September 22, 2010 (2010/63/EU) and with the Italian D.L. n. 26/2014 and were approved by the Local Committee for Animal Care and Welfare of the University of Genova and the Italian Ministry of Health (DDL 26/2014 and previous legislation; protocol number n° 75F11.N.IMY). All efforts were made to minimize the number of animals used and their suffering, and no *in vivo* technique was used.

### Preparation of Synaptosomes

**FIGURE 1:**
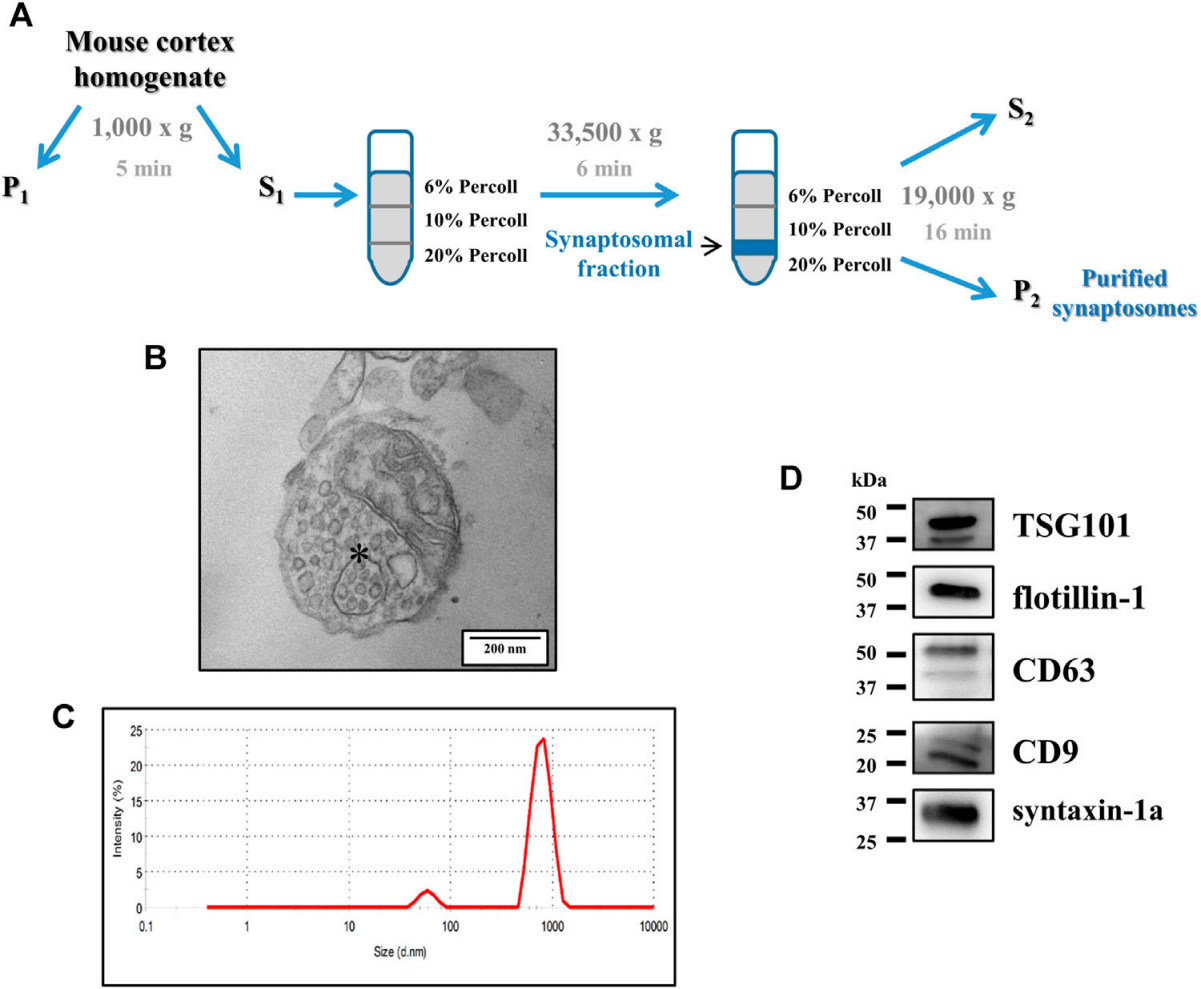
Structural analysis of purified synaptosomes isolated from the mouse cortex. **(A)** Schematic representation of the discontinuous Percoll gradient isolation procedure of the purified synaptosomes from the mouse cortical homogenate. For further details, see the Methods section. **(B)** Ultrastructural analysis of synaptosomes by transmission electron microscopy (TEM). The figure shows an isolated synaptosome, containing multiple vesicles, a mitochondrion, and a multivesicular body–like organelle (asterisk). Scale bar: 200 nm. The image is representative of the TEM analysis of *n* = 5 different synaptosomal preparations. **(C)** Size distribution of synaptosomes assessed using a Zetasizer Nano ZS90 particle sizer. The curve is representative of the analysis of *n* = 5 different synaptosomal preparations. **(D)** Western blot analysis of the exosomal markers TSG101, flotillin-1, CD63, and CD9 in cortical synaptosomal lysates (5 μg/lane). Syntaxin-1a was used as a synaptosomal marker. The image is representative of the analysis of *n* = 6 synaptosomal lysates.

Synaptosomes can be easily obtained by homogenization of brain tissues, their membranes having “pinched off” at the point of connection with the neuronal axon, and then purified from other tissue components using density-gradient centrifugation techniques (Gray and Wittaker, 1962; Dunkley et al., 2008). Purified synaptosomes were prepared as previously described (Olivero et al., 2018) from the cortex of one animal/experiment, except for the experiments described in Figure 4, where the cortices of two mice/experiments were pooled together. The tissue was homogenized in 10 volumes of 0.32 M sucrose and buffered to a pH value of 7.4 with Tris (final concentration 0.01 M) using a glass/Teflon tissue grinder (clearance 0.25 mm). The homogenate was first centrifuged at 1,000 g for 5 min to remove nuclei and debris; the supernatant was gently layered on a discontinuous Percoll gradient (6, 10, and 20% v/v in Tris-buffered 0.32 M sucrose) and then centrifuged at 33,500 g for 6 min. The layer between 10 and 20% Percoll (synaptosomal fraction) was collected and washed by centrifugation at 19,000 g for 15 min (Figure 1A). Synaptosomes were then resuspended in a physiological medium having the following composition (mM): NaCl, 140; KCl, 3; MgSO_4_, 1.2; CaCl_2_, 1.2; NaH_2_PO_4_, 1.2; NaHCO_3_, 5; HEPES, 10; glucose, 10; pH 7.4.

### Depolarization and Pharmacological Treatments

The synaptosomal suspension was then divided into identical aliquots and preincubated for 5 min in the physiological medium in a water bath at 37°C under mild shaking to equilibrate the system. Synaptosomal aliquots were then incubated for 2 min with the physiological medium containing 3 mM KCl (3 mM KCl medium, basal condition, control synaptosomes) or with a KCl-enriched solution (25 mM KCl-containing medium, NaCl substituted for an equimolar amount of KCl, depolarized synaptosomes). At t = 2 min, cold 3 mM KCl medium was added to the reaction tubes to stop the incubation, and the synaptosomal suspensions were centrifuged at 19,000 g for 10 min to separate the synaptosomal pellets from the supernatants.

When indicated, synaptosomes were incubated for 2 min with a 25 mM KCl medium lacking Ca^2+^ ions (25 mM KCl/Ca^2+^-free medium) or with a 25 mM KCl medium containing the GABA_B_ receptor agonist (±)-baclofen (10 μM, Tocris Bioscience, Bristol, United Kingdom).

The synaptosomal pellets were quantified for the protein content using Pierce BCA assay (Thermo Fisher Scientific, Waltham, MA, United States) and then lysed in modified RIPA buffer (10 mM Tris, pH 7.4, 150 mM NaCl, 1 mM EDTA, 0.1% SDS, 1% Triton X-100, protease inhibitors) for Western blot analysis or resuspended in PBS for transmission electron microscopy and dynamic light scattering analyses. The synaptosomal supernatants were centrifuged at 19,000 g for 30 min to remove any synaptosomal debris and collected for the isolation of exosomes.

### Isolation of the Exosomes From the Synaptosomal Supernatants

Identical volumes of the synaptosomal supernatants from the different experimental conditions were incubated with the Total Exosome Isolation Reagent (from cell culture media, Invitrogen, Thermo Fisher Scientific) overnight at 4°C and then centrifuged at 10,000 g for 1 h at 4°C. Pellets corresponding to the exosomal fractions were collected and resuspended in modified RIPA lysis buffer for the Western blot analysis or in PBS for flow cytometry, transmission electron microscopy, and dynamic light scattering analyses.

### Western Blot Analysis

The exosomal lysates from the different experimental conditions were dissolved in SDS-PAGE sample buffer, boiled for 5 min at 95°C, subjected to 10% SDS-PAGE, and then blotted onto PVDF membranes (Merck, Darmstadt, Germany). Membranes were blocked for 1 h at room temperature with Tris-buffered saline-Tween (t-TBS: 20 mM Tris, pH 7.4, 150 mM NaCl, and 0.05% Tween 20), containing 5% (w/v) nonfat dried milk, and then incubated overnight at 4°C with the following primary antibodies: rabbit anti-TSG101 (1:500, T5701, Sigma-Aldrich, St. Louis, MO, United States), rabbit anti–flotillin-1 (1:1,000, 18634, Cell Signaling Technology, Danvers, MA, United States), rabbit anti-CD63 (1:1,000, sc-5275, Santa Cruz Biotechnology, Dallas, TX, United States), and rabbit anti-CD9 (1:300, ab92726, Abcam, Cambridge, United Kingdom). After extensive washes in t-TBS, membranes were incubated for 1 h at room temperature with appropriate horseradish peroxidase–linked secondary antibodies (1:10,000, A9044 and A9169, Sigma-Aldrich).

Also the cortical synaptosomal lysates (5 µg/lane) were analyzed with immunoblot analysis, and membranes were probed with the primary antibodies mentioned above (concentrations as previously indicated) and with mouse anti–syntaxin-1a antibody (1:500, GTX18010, GeneTex, Irvine, CA, United States).

In a set of Western blot experiments, an equal amount of the synaptosomal and exosomal lysates (2 µg/lane) were compared for the content of selected proteins by using the primary antibodies mentioned already, at the concentrations indicated, and the following ones: mouse anti–synaptotagmin-1 (1:500, 105 011, Synaptic System, Goettingen, Germany) and rabbit anti-PSD95 (1:1,000, NBP1-40474, Novus Biologicals, Centennials, CO, United States).

Immunoblots were visualized using the enhanced chemiluminescence Western blotting detection system Immobilon Forte Western HRP substrate (Merck). Images were acquired by using an Alliance LD6 image capture system (Uvitec, Cambridge, United Kingdom) and analyzed using UVI-1D software (Uvitec).

### Transmission Electron Microscopy

Freshly prepared synaptosomes were washed out in 0.1 M cacodylate buffer and immediately fixed in 0.1 M cacodylate buffer containing 2.5% glutaraldehyde (Electron Microscopy Science, Hatfield, PA, United States) for 1 h at room temperature. The synaptosomes were postfixed in osmium tetroxide for 2 h and 1% uranyl acetate for 1 h. Subsequently, samples were dehydrated through a graded ethanol series and embedded in resin (Poly-Bed; Polysciences, Inc., Warrington, PA, United States) for 24 h at 60°C. Ultrathin sections (50 nm) were cut and stained with 5% uranyl acetate in 50% ethanol.

Electron microscopic analysis on isolated vesicle preparations was performed as follows. The extracellular vesicle preparations were resuspended in 20 μL PBS (pH 7.4) and fixed by adding an equal volume of 2% paraformaldehyde in 0.1 mol/L phosphate buffer (pH 7.4). Extracellular vesicles were then adsorbed for 10 min onto formvar–carbon–coated copper grids by floating the grids on 5 μl drops on parafilm. Subsequently, grids with adhered vesicles were rinsed in PBS and negatively stained with 2% uranyl acetate for 5 min at room temperature. Stained grids were embedded in 2.5% methylcellulose for improved preservation and air-dried before examination (Cortese et al., 2020). Electron micrographs were taken using a Hitachi TEM microscope (HT7800 series, Tokyo, Japan) equipped with a Megaview 3 digital camera and Radius software (EMSIS, Germany).

### Dynamic Light Scattering and Zeta-Potential

Particle size, polydispersity, and zeta-potential were analyzed using a Zetasizer Nano ZS90 particle sizer at a fixed angle of 90° (Malvern Instruments, Worchestershire, United Kingdom). Vesicle size was evaluated by dynamic light scattering, allowing the analysis of particles within the range of 0.1–10◦μm, as previously described (Marimpietri et al., 2013). Briefly, exosomes and synaptosomes were suspended in PBS, and the measure was performed at a constant temperature of 25°C in UV–transparent cuvettes. The translational diffusion coefficient of the solutions was calculated from the time autocorrelation of the scattered light intensity, and the translational diffusion coefficient was extracted from the correlogram using the method of cumulants as applied in proprietary Malvern software. The diameter of the exosomes was obtained from the application of the Stokes–Einstein equation as follows: d(H) = kT/3 pgD, where d(H) is the hydrodynamic diameter, k the Boltzmann constant, T the temperature, g the shear viscosity of the solvent, and D the diffusion coefficient of the particles.

### Flow Cytometric Analysis

Exosomes were analyzed for the presence of CD9 and TSG101 by flow cytometry after vesicles adsorption onto latex beads as previously reported (Marimpietri at al., 2013). In brief, 1 ml of purified exosomes was incubated with 4 μl of aldehyde/sulfate latex beads with a diameter of 4 mm (Invitrogen, Thermo Fisher Scientific) for 2 h at room temperature; PBS supplemented with 4% fetal bovine serum was then added to each sample, and the incubation was prolonged for 30 min. After washing, exosome-coated beads were incubated for 30 min at 4°C with primary rat anti-mouse CD9 PE-conjugated monoclonal antibody (12-0091-81, Invitrogen, Thermo Fisher Scientific) or with unlabeled mouse anti-TSG101 monoclonal antibody (ab43, Abcam), plus further incubation for 20 min at 4°C with a goat anti-mouse PE-conjugated secondary antibody (PA5-33249, Thermo Fisher Scientific). As negative control, an isotype-matched primary monoclonal antibody was used. All antibodies were used in accordance with the manufacturer’s instructions. Samples were then analyzed using a Gallios flow cytometer and Kaluza software (BeckmanCoulter, Brea, CA, United States).

### Quantification of the Endogenous Glutamate Content

The endogenous glutamate content in the synaptosomal supernatants was analyzed by high-performance liquid chromatography analysis after precolumn derivatization with ophthalaldehyde and separation on a C18 reverse-phase chromatographic column (10 mm × 4.6 mm, 3 μm; at 30°C; Chrompack, Middleburg, Netherlands) coupled with fluorimetric detection (excitation wavelength, 350 nm; emission wavelength, 450 nm). Buffers and the gradient program were previously described (Salamone et al., 2014). Homoserine was used as the internal standard. The amount of glutamate was expressed as pmol/mg of synaptosomal proteins.

### LDH Assay

The LDH activity in the synaptosomal supernatants was evaluated by using Pierce LDH Cytotoxicity Assay Kit (Thermo Fisher Scientific) according to the manufacturer’s manual. The LDH activity in the supernatants was expressed as percent of the total synaptosomal LDH activity (LDH activity in synaptosomal pellets and in the supernatants).

### Experimental Design and Statistical Analysis

Data represent the mean ± SEM of n independent replications (n indicated in the figure legends). Sigma Plot 10 data analysis and the graphing software package was used for data handling/statistics and for graph drawing. Analysis of variance was performed using ANOVA, followed by Tukey’s multiple-comparisons test; direct comparisons were performed using Student’s *t*-test. Data were considered significant if *p* < 0.05, at least.

## Results

### Cortical Synaptosomes Possess Proteins That Are Markers of Exosomes

**FIGURE 2:**
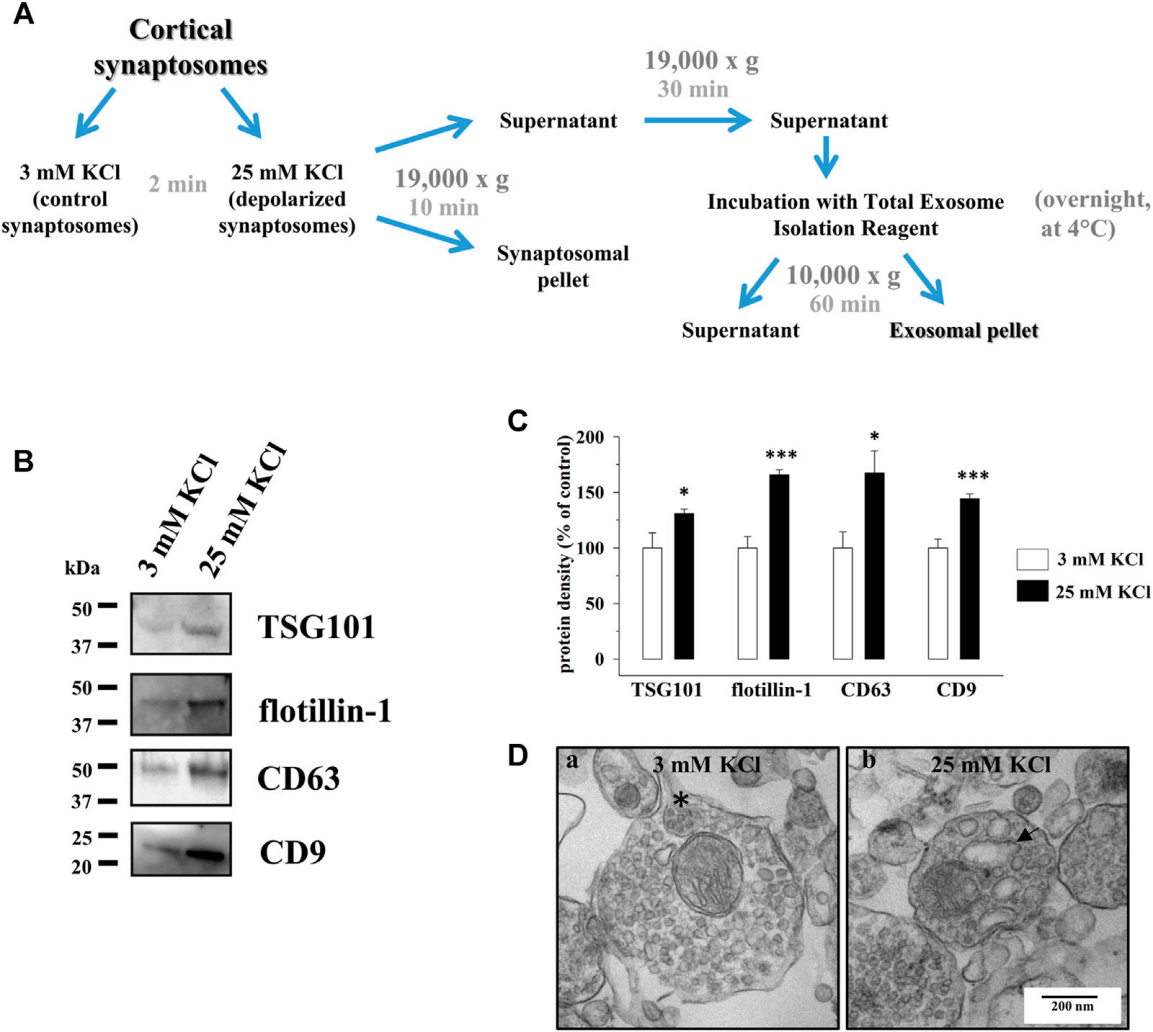
Cortical synaptosomes release extracellular vesicles immunopositive for the exosomal markers TSG101, flotillin-1, CD63, and CD9, and their secretion is increased by depolarization. **(A)** Schematic representation of the isolation procedure of the exosomes from the supernatants of the synaptosomes incubated for 2 min in 3 mM KCl medium (control synaptosomes) or in 25 mM KCl medium (depolarized synaptosomes). For further details, see the Methods section. **(B)** Western blot analysis of TSG101, flotillin-1, CD63, and CD9 proteins in the exosomal fractions isolated from the supernatants of the control (lane 3 mM KCl) and depolarized synaptosomes (lane 25 mM KCl). The image is representative of the results of *n* = 5 (TSG101), *n* = 6 (CD63), and *n* = 11 (flotillin-1 and CD9) Western blot analyses. **(C)** Quantification of the change in TSG101, flotillin-1, CD63, and CD9 proteins in the exosomal fraction from the supernatants of depolarized synaptosomes (25 mM KCl, black bars) when compared to the control synaptosomes (3 mM KCl, empty bars). Results are expressed as percent of control (3 mM KCl). Data represent the mean ± SEM. *p < 0.05 vs. respective control; ***p < 0.001 vs. respective control. **(D)** Ultrastructural analysis of the control and depolarized cortical synaptosomes by TEM. The figure shows a representative image of synaptosomes incubated for 2 min in 3 mM KCl medium (a) and in 25 mM KCl medium (b). Asterisk, MVB-like organelle in control synaptosomes; arrow, endosomal-like organelle in depolarized synaptosomes. Scale bar: 200 nm. The image is representative of *n* = 5 different synaptosomal preparations.

Purified cortical synaptosomes were analyzed at the ultrastructural level by transmission electron microscopy (TEM). Consistent with previous data in the literature (Feligioni et al., 2006; Nisticò et al., 2015), synaptosomes appeared as round-shaped membrane-bound structures having a diameter in the range of 500–1,200 nm, containing intraterminal mitochondria, small clathrin-coated vesicles, and abundant synaptic vesicles. Some of them were endowed with endosome-like structures, including multivesicular body (MVB)-like organelles, with intraluminal vesicles (Figure 1B but also Figure 2Da).

Cortical synaptosomes were also analyzed by dynamic light scattering, which confirmed the size range observed in the TEM analysis, with a bell-shaped size distribution profile of the synaptosomal particles peaking at 790 ± 67 nm (Figure 1C).

The presence of MVB-like organelles in the purified synaptosomes prompted us to investigate the presence of proteins widely recognized as exosomal markers: the endosomal sorting complex required for transport I tumor susceptibility gene 101 protein (TSG101), the lipid raft-associated protein flotillin-1, and the tetraspanins CD63 and CD9 (Andreu and Yànez-Mò, 2014; Zhang et al., 2019). The Western blot analysis unveiled a clear immunoreactivity having appropriate mass for all the proteins mentioned above (expected molecular weights: TSG101, 46 kDa; flotillin-1, 49 kDa; CD63, 30–60 kDa depending on the glycosylated forms of the protein; CD9, 25 kDa; Figure 1D).

### Cortical Synaptosomes Exposed to a Depolarizing Stimulus Release Extracellular Vesicles Having the Biochemical and Morphological Features of Exosomes

Based on the presence of MVB-like organelles in cortical synaptosomes and on the immunoreactivity for the exosomal markers TSG101, flotillin-1, CD9, and CD63 in the synaptosomal lysates, we asked whether exosomes can also be actively released from axonal terminals.

To test the hypothesis, identical aliquots of cortical synaptosomes were incubated under basal, non-depolarizing condition (3 mM KCl medium, control synaptosomes) or in the presence of a mild depolarizing stimulus (25 mM KCl medium, depolarized synaptosomes). Extracellular vesicles (EVs) that had the dimension and the features of exosomes were then isolated by means of the Total Exosome Isolation Reagent from identical volumes of the synaptosomal supernatants (Figure 2A, see also the Methods section) and analyzed for the presence of the exosomal markers listed above: TSG101, flotillin-1, CD63, and CD9. The Western blot analysis unveiled that the EV fractions isolated from the supernatants of both the control and depolarized synaptosomes were immunopositive for all the proteins (Figure 2B). A significant increase in the TSG101, flotillin-1, CD63, and CD9 immunoreactivities in the fraction from the depolarized synaptosomes emerged when compared to the control (Figure 2C). Concomitantly, the comparative ultrastructural analysis by TEM of the control and depolarized cortical synaptosomes unveiled that depolarized synaptosomes were almost devoid of MVB-like organelles when compared to the control ones but contained empty structures that Leenders et al. (2002) defined as endosomal-like organelles (Leenders et al., 2002; Figure 2Db).

**FIGURE 3:**
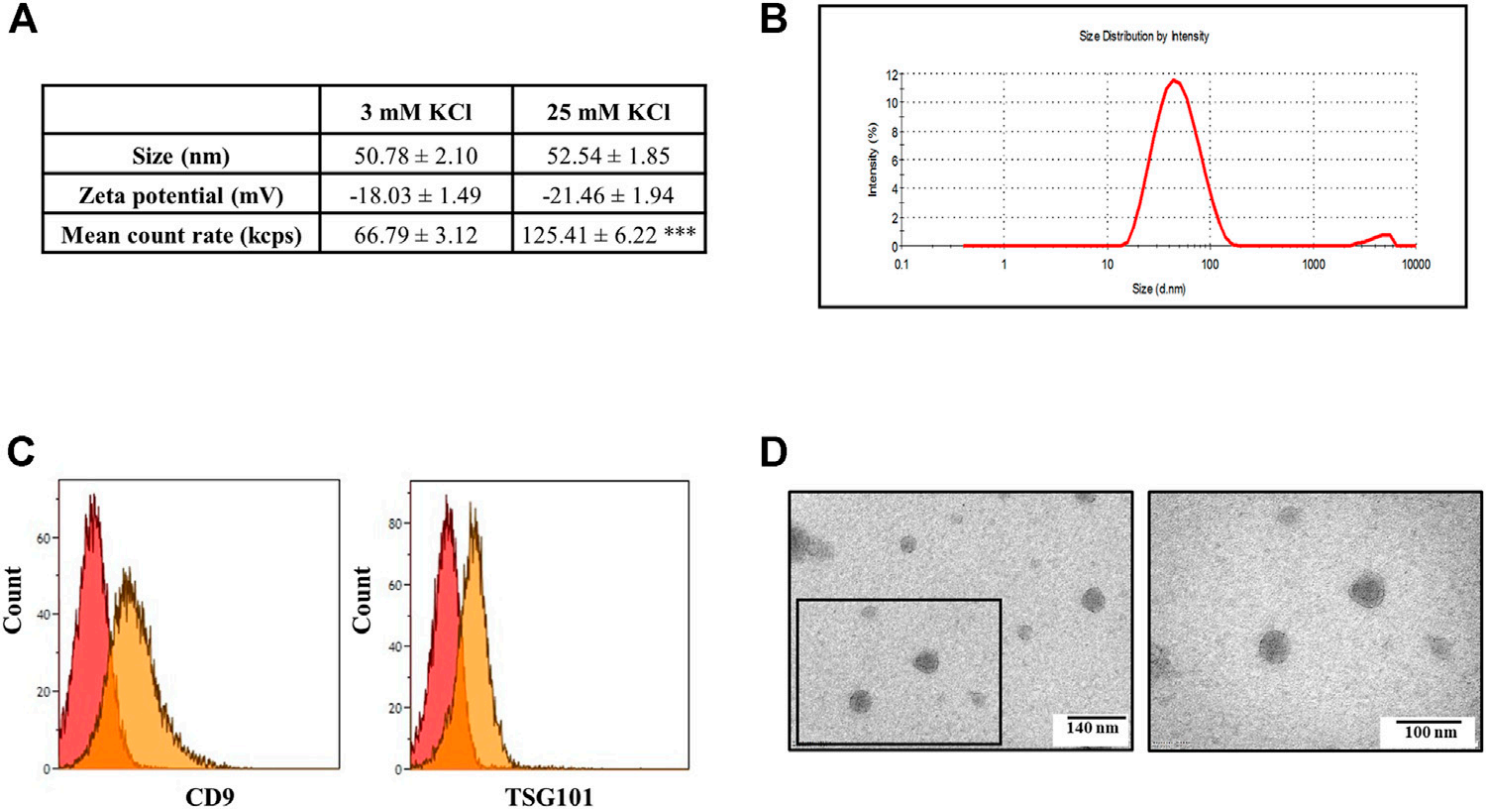
Characterization of the exosomes released from cortical synaptosomes by light dynamic scattering, flow cytometry, and transmission electron microscopy (TEM). **(A)** Size (nM), zeta-potential (mV), and mean count rate (kcps) values detected using a Zetasizer Nano ZS90 particle sizer during the analysis of the exosomes isolated from the supernatants of the control (3 mM KCl) and depolarized synaptosomes (25 mM KCl). Data represent the mean ± SEM of *n* = 5 (control synaptosomes) and *n* = 7 (depolarized synaptosomes) analyses. ***p < 0.001 vs. the mean count rate value of 3 mM KCl. **(B)** Size distribution of the exosomes assessed using a Zetasizer Nano ZS90 particle sizer. The curve is representative of the analysis of *n* = 8 exosomal preparations. **(C)** Flow cytometric analysis of TSG101 and CD9 expression in the exosomes purified from the supernatants of depolarized synaptosomes and coated on latex beads. Images are representative of the analysis of *n* = 5 exosomal preparations. **(D)** Negative-stained exosomes isolated from the supernatants of depolarized synaptosomes observed by TEM. The left image shows the round and smooth morphology of isolated exosomes, and the right picture represents an enlargement (squared box) of the original image. The electron micrographs are representative of *n* = 5 exosomal preparations. Scale bar right: 140 nm; scale bar left: 100 nm.

The EVs isolated from the synaptosomal supernatants were then analyzed by dynamic light scattering for their size and zeta-potential. The size distribution profile of the EVs isolated from the depolarized synaptosomes did not differ significantly from that of the EVs isolated from the control synaptosomes. Both curves showed a bell-shaped profile that reached the peak at 50.78 ± 2.10 nm for the EVs isolated from the supernatants of control synaptosomes and at 52.54 ± 1.85 nm for the EVs isolated from the supernatants of depolarized synaptosomes (Figures 3A,B). The size range for both profiles was consistent with the expected size values for exosomes. Furthermore, the EVs from both control and depolarized synaptosomes showed a comparable zeta-potential (Figure 3A), suggesting a good and similar nanoparticle stability in terms of dispersion, aggregation, or flocculation in both preparations. However, the mean count rate of dynamic light scattering (the parameter that represents the average scattering intensity during the measurement) was almost double (125.41 ± 6.22 kcps) when analyzing the EVs isolated from the supernatants of depolarized synaptosomes when compared to that recorded during the analysis of the EVs from the supernatants of the control synaptosomes (66.79 ± 3.12 kcps, Figure 3A). Since the mean count rate is directly proportional to the concentration of the EVs in the analyzed sample, the data indirectly imply that the EVs isolated from the supernatants of the control and depolarized synaptosomes differ quantitatively but not qualitatively.

The latex bead cytofluorimetric assay of the EVs isolated from the supernatants of the 25 mM KCl-depolarized synaptosomes confirmed good levels of signals for CD9 and TSG101, two of the most typical exosomal surface markers (Figure 3C). Furthermore, TEM analysis demonstrated that the EVs released from the synaptosomes after depolarization have a round shape and a smooth surface, with a diameter ranging from 40 to 70 nm, which is consistent with the exosomal ultrastructure (Figure 3D). Based on these observations, the term “exosomes” will be used from here on out to indicate the released EVs.

### Comparative Analysis of Exosomal and Synaptosomal Markers in Synaptosomal and Exosomal Lysates

**FIGURE 4:**
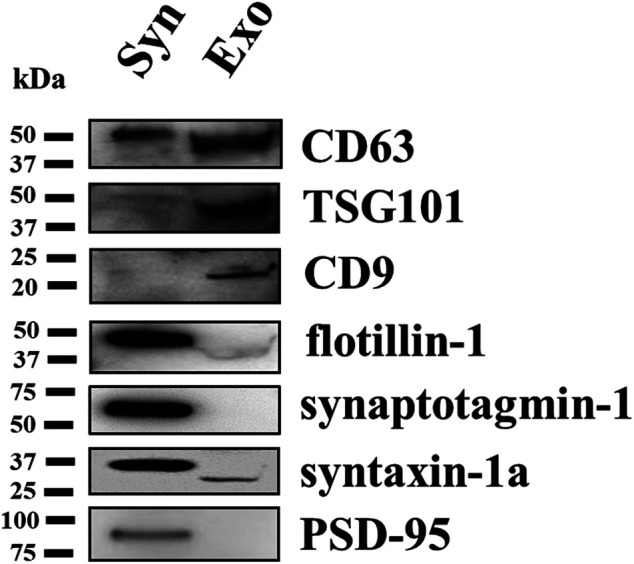
Comparative Western blot analysis of selected proteins in the cortical synaptosomal and exosomal lysates. Identical amounts (2 μg proteins/lane) of the cortical synaptosomal lysate (lane Syn) and of the exosomal lysate (lane Exo) were loaded on 10% SDS-PAGE and analyzed for the contents of the exosomal markers TSG101, CD63, CD9, and flotillin-1, and of the synaptosomal markers synaptotagmin-1, syntaxin-1a, and PSD-95. The image is representative of the Western blot analysis on *n* = 5 synaptosomal and exosomal preparations.

Western blot analysis was carried out to compare the content of selected synaptosomal and exosomal markers in the lysates of the exosomes and of the depolarized synaptosomes they are released from. Identical amounts of proteins (2 μg/lane) of both lysates were analyzed for the exosomal markers TSG101, flotillin-1, CD63, and CD9. The exosomal lysates were particularly enriched in the TSG101, CD63, and CD9 proteins when compared to the synaptosomal ones, while flotillin-1 was expressed in synaptosomes and to a lesser extent in the exosomal fraction. We then focused on the following synaptic markers: synaptotagmin-1, a synaptic vesicle membrane protein; syntaxin-1a, which is located in presynaptic plasma membranes; and the postsynaptic density protein 95 (PSD-95), a membrane protein that has a postsynaptic localization (Bonanno et al., 2005; Bonfiglio et al., 2019; Franchini et al., 2019). The analysis unveiled that these proteins are present (although to a different level, PSD-95 showing a scarce immunoreactivity) in the synaptosomal lysates (expected protein weight: synaptotagmin-1, 60 kDa; syntaxin-1a, 36 kDa; PSD-95, 95 kDa) but not in the exosomal lysates where only a slight immunopositivity for syntaxin-1a was observed (Figure 4). These observations unveiled a qualitative difference in the protein composition of the synaptosomal and the exosomal preparations.

### The Release of Exosomes From Cortical Synaptosomes is a Ca^2+^-Dependent Event

**FIGURE 5:**
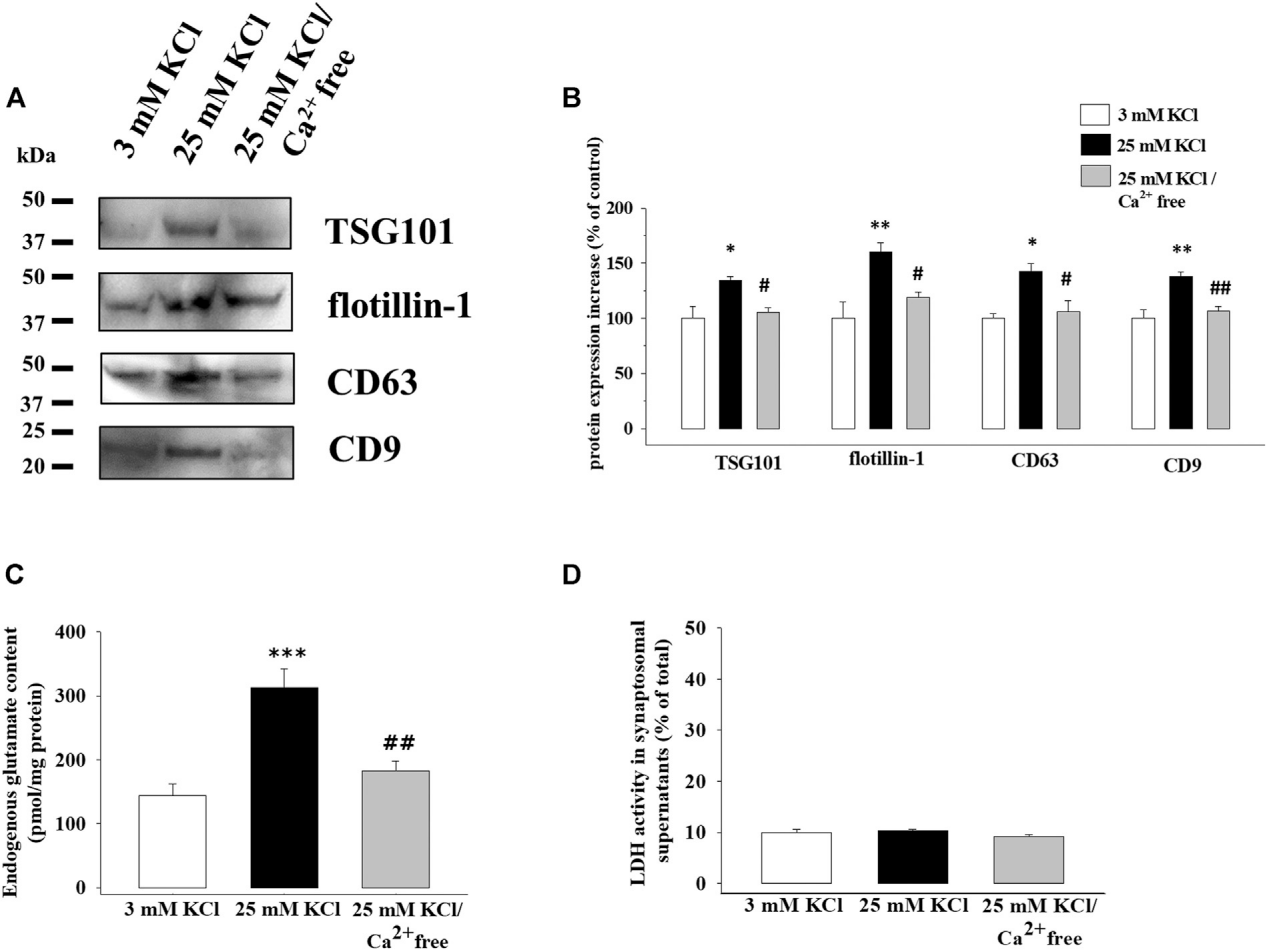
Release of exosomes from cortical synaptosomes is a Ca^2+^-dependent event. Synaptosomes were incubated for 2 min in basal condition, in a 25 mM KCl medium containing 1.2 mM Ca^2+^ ions or lacking Ca^2+^ ions (Ca^2+^ free medium). **(A)** Western blot experiments were carried out to analyze TSG101, flotillin-1, CD63, and CD9 protein content in the exosomal fractions isolated from the supernatants of the synaptosomes under the three experimental conditions (respectively, lane 3 mM KCl, lane 25 mM KCl, and lane 25 mM KCl/Ca^2+^ free). The image is representative of the results of *n* = 4 (TSG101), *n* = 5 (CD63), *n* = 6 (CD9), and *n* = 8 (flotillin-1) Western blot analyses. **(B)** Quantification of the change in TSG101, flotillin-1, CD63, and CD9 proteins in the exosomal fraction from the supernatants of the synaptosomes exposed to 25 mM KCl/Ca^2+^-free medium (gray bars) when compared to 25 mM KCl/1.2 mM Ca^2+^ medium (25 mM KCl, black bars). Results are expressed as percent of control (3 mM KCl, empty bars). Data represent the mean ± SEM. *p < 0.05 vs. respective control; **p < 0.01 vs. respective control; ^#^ *p* < 0.05 vs. 25 mM KCl; ^##^ *p* < 0.01 vs. 25 mM KCl. **(C)** Endogenous glutamate content in the supernatants of the synaptosomes incubated in 3 mM KCl medium (empty bar), 25 mM KCl/1.2 mM Ca^2+^-containing medium (25 mM KCl, black bar), and 25 mM KCl/Ca^2+^-free medium (gray bar). The endogenous glutamate content is expressed as pmol/mg synaptosomal proteins. Data represent the mean ± SEM of the analysis of *n* = 11 samples. ***p < 0.001 vs. 3 mM KCl; ^##^ *p* < 0.01 vs. 25 mM KCl. **(D)** Lactate dehydrogenase (LDH) activity in the supernatants of the synaptosomes incubated in 3 mM KCl medium (empty bar), 25 mM KCl/1.2 mM Ca^2+^-containing medium (25 mM KCl, black bar), and 25 mM KCl/Ca^2+^-free medium (gray bar). The LDH activity in the supernatants is expressed as percent of total LDH activity (LDH activity in the synaptosomal pellets and supernatants). Data represent the mean ± SEM of the analysis of *n* = 5 samples.

Identical aliquots of cortical synaptosomes were exposed to the 25 mM KCl medium containing a physiological amount of Ca^2+^ ions (1.2 mM Ca^2+^-containing medium) or to the 25 mM KCl medium lacking the divalent cation (Ca^2+^-free medium). As shown in the representative Western blot in Figure 5A, a significant reduction in TSG101, flotillin-1, CD63, and CD9 protein content was observed in the exosomal fraction isolated from the supernatants of synaptosomes exposed to the 25 mM KCl/Ca^2+^-free medium when compared to synaptosomes depolarized with the 25 mM KCl/1.2 mM Ca^2+^-containing medium (Figure 5B).

As expected, the endogenous glutamate content in the supernatants from synaptosomes exposed to the 25 mM KCl/1.2 mM Ca^2+^-containing medium was significantly increased when compared to that from synaptosomes under basal condition (3 mM KCl), but the omission of calcium ions in the external medium almost totally prevented the releasing activity (Figure 5C).

To exclude the possibility that a nonspecific synaptosomal leakage might account for the results described so far, we verified the viability of the synaptosomes under the different experimental conditions by measuring the endogenous lactate dehydrogenase (LDH) activity in the supernatants. The LDH activity was unmodified in the supernatants of the control and depolarized synaptosomes, and also the removal of external Ca^2+^ ions from the 25 mM KCl medium did not affect this parameter (Figure 5D).

### The Release of Exosomes From Cortical Synaptosomes is not Modulated by Presynaptic GABA_B_ Heteroreceptors

**FIGURE 6:**
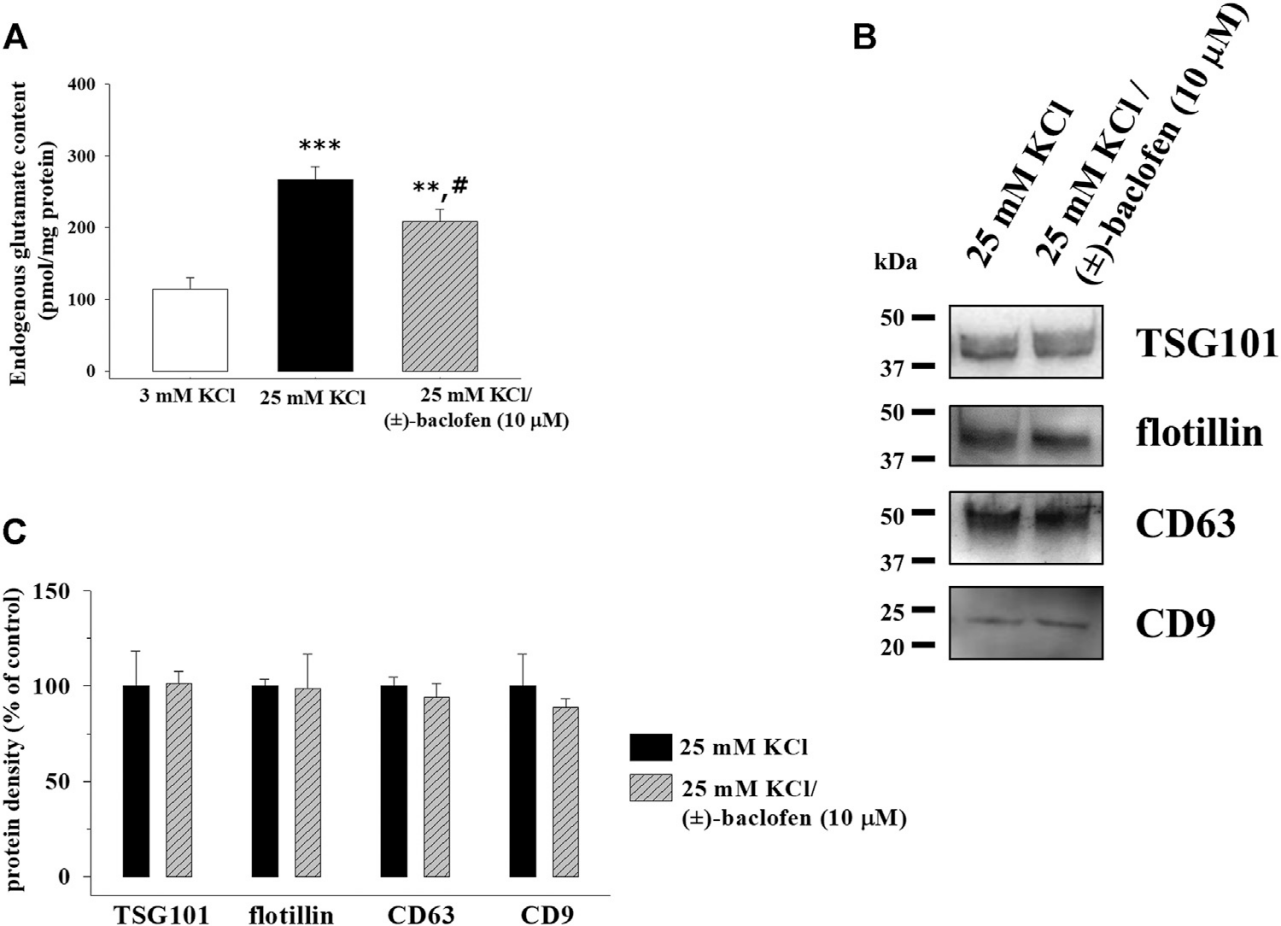
Release of exosomes from cortical synaptosomes is not modified by presynaptic GABA_B_ receptor activation. Synaptosomes were incubated for 2 min in a 25 mM KCl medium in the absence or in the presence of the GABA_B_ receptor agonist (±)-baclofen (10 μM). **(A)** Endogenous glutamate content in the supernatants of the synaptosomes incubated in 3 mM KCl medium (empty bar), 25 mM KCl medium (black bar), and 25 mM KCl/(±)-baclofen (10 μM) medium (rising right hatched gray bar). The endogenous glutamate content is expressed as pmol/mg synaptosomal proteins. Data represent the mean ± SEM of the analysis of *n* = 12 samples. **p < 0.01 vs. control; ***p < 0.001 vs. control; ^#^ *p* < 0.05 vs. 25 mM KCl. **(B)** Western blot experiments were carried out to analyze TSG101, flotillin-1, CD63, and CD9 protein content in the exosomal fractions isolated from the supernatants of synaptosomes stimulated with 25 mM KCl alone (lane 25 mM KCl) or in the presence of (±)-baclofen (10 μM) (lane 25 mM KCl/(±)-baclofen (10 μM)). The image is representative of the results of *n* = 4 (TSG101), *n* = 6 (CD63), *n* = 9 (flotillin-1), and *n* = 10 (CD9) Western blot analyses. **(C)** Quantification of the change in TSG101, flotillin-1, CD63, and CD9 proteins in the exosomal fraction from the supernatants of the synaptosomes exposed to 25 mM/(±)-baclofen (10 μM) medium (rising right hatched gray bars) when compared to 25 mM KCl medium (black bars). Results are expressed as percent of control (25 mM KCl).

Glutamate exocytosis from cortical synaptosomes is an active process tuned by auto- and heteroreceptors presynaptically located in nerve terminals. In particular, presynaptic inhibitory GABA_B_ heteroreceptors exist in cortical glutamatergic nerve endings, whose activation reduces the adenylyl cyclase activity and the Ca^2+^ conductance, inhibiting glutamate exocytosis from the nerve endings (Bonanno and Raiteri., 1993; Grilli et al., 2004; Vergassola et al., 2018; Pittaluga 2019). Accordingly, the glutamate content in the supernatants of synaptosomes exposed to 25 mM KCl medium in the presence of the GABA_B_ receptor agonist (±)-baclofen (10 µM) was significantly lower than that in the supernatants of synaptosomes exposed to the depolarizing stimulus alone (Figure 6A). Differently, the amount of TSG101, flotillin-1, CD63, and CD9 in the exosomal fraction isolated from the synaptosomes exposed to the depolarizing stimulus in the presence of (±)-baclofen (10 µM) did not significantly differ from that in the exosomal fraction isolated from synaptosomes exposed to the depolarizing stimulus alone (Figures 6B,C).

## Discussion

In recent years, exosomes have emerged as nonconventional messengers in intercellular communication in the CNS, and their role in the modulation of synaptic activity in physiological and pathological conditions has gained interest (Smalheiser, 2007; Saeedi et al., 2019; Schiera et al., 2020).

Although MVBs, the late endosomal organelles from which exosomes originate and are released from, are less present in central axonal terminals than in other neuronal compartments (i.e., somata and dendrites; Von Bartheld and Altick, 2011; Men et al., 2019), some studies supported their presynaptic location and the possibility that they can fuse with nerve terminal plasma membranes (Koles et al., 2012; Korkut et al., 2013; Janas et al., 2016 and references therein; Zappulli et al., 2016; Zhang and Yang, 2018). Although these findings would support the presence and the release of exosomes from nerve endings, clear evidence demonstrating this conclusion is lacking. With the aim of answering this question, we focused on synaptosomes (Pittaluga, 2019), convinced that this preparation represents an appropriate model to study the presynaptic events. Synaptosomes are pinched-off nerve terminals (see Figure 1B). Which retain the main features of the synaptic boutons they originate from and contain structures with a presynaptic origin that account for the main presynaptic functional events (e.g., the uptake, the synthesis, the metabolism, the storage, and the release of transmitters). Synaptosomes are also endowed with naïve proteins and receptors, controlling these presynaptic functions, including the release of neurotransmitters. In a few cases, synaptosomes retain fragments of the postsynaptic membranes that remain attached to the presynaptic side by means of proteins (e.g., PSD95) but that do not reseal and cannot influence the functional synaptosomal responses (Raiteri and Raiteri, 2000).

The first finding of this study is that mouse cortical synaptosomal lysates are immunopositive for endosome-associated proteins, commonly considered as exosomal markers (i.e., TSG101, flotillin-1, CD63, and CD9), consistent with the presence of exosomes in isolated nerve endings. In line with the conclusion, the results from TEM ultrastructural analysis unveiled the presence of MVB-like organelles containing intraluminal vesicles in cortical synaptosomes.

Second, we demonstrated that mouse cortical synaptosomes release EVs with the structural and biochemical features of exosomes in response to a depolarizing stimulus. The conclusion relies on the following main findings:

1) the supernatants of cortical synaptosomes exposed to a mild depolarizing stimulus (25 mM KCl medium) were enriched with EVs that, in both dynamic light scattering and TEM analyses, displayed a size and shape consistent with those of exosomes.
2) the size and the zeta-potential of the EVs isolated from the supernatants of synaptosomes under both basal and depolarized conditions were largely conserved, but a significant increase in the mean count rate of dynamic light scattering was observed when analyzing EVs from depolarized terminals. We considered that the latter observation might be predictive of an accumulation of EVs at the outer side of synaptosomes upon the application of the depolarizing stimulus.
3) the EV fraction isolated from the supernatants of depolarized synaptosomes were more reactive for TSG101, flotillin-1, CD63, and CD9 than the EV fraction collected under the basal condition.
4) MVB-like organelles were rarely, if ever, detected in depolarized synaptosomes when compared to control synaptosomes, which might suggest the depletion of these organelles in depolarized nerve terminals because of the increased release of exosomes.

The comparative analysis of synaptosomal and exosomal proteins in identical amounts of synaptosomal and exosomal lysates unveiled a different proteomic profile of the two preparations, stressing that synaptosomes and exosomes are different in nature. Specifically, as far as the exosomal markers are concerned, the exosomal lysates were particularly enriched in the TSG101, CD9, and CD63 proteins. Differently, flotillin-1 was particularly expressed in the synaptosomal lysates and to a lower extent in the exosomal preparation. This is not surprising, since this protein concentrates within the cholesterol-enriched microdomains of the lipid rafts, which are also components of the neuronal plasma membranes, to control the synapsis formation (Swanwick et al., 2010).

Turning to the synaptosomal markers, synaptotagmin-1, a v-SNARE protein located in vesicular membranes, was largely expressed in synaptosomal lysates but absent in the exosomal fraction, which would exclude a contamination of synaptic vesicles in the exosomal preparation. Similarly, the lack of PSD95 immunopositivity, a specific marker for the postsynaptic compartment, in the exosomal preparation excludes the presence of postsynaptic impurities and the possibility that exosomes could originate from the postsynaptic membrane fragments, if present. Differently, syntaxin-1a, a t-SNARE membrane protein mediating vesicular exocytosis, was slightly detected in the exosomal lysates. This would reflect a contamination since, at least in the *Drosophila* neuromuscular junction (Koles et al., 2012), the protein assures the anchoring of MVBs to plasma membranes, participating in the membrane–membrane interaction that drives the docking and the fusion of MVBs and the consequent release of exosomes.

Another interesting observation of the study is that the amount of exosomal markers in the exosomal fraction isolated from the supernatants of depolarized synaptosomes was drastically reduced when exposing the nerve endings to the 25 mM KCl/Ca^2+^-free medium. The finding is in line with previous results already present in the literature (Savina et al., 2003; Krämer-Albers et al., 2007; Lachenal et al., 2011) and supports the conclusion that the release of exosomes is an active event which depends on triggering events linked to the influx of the divalent cation in the nerve terminals.

The influx of calcium ions is pivotal to several processes in nerve terminals, including the exocytosis of transmitters, which largely depends on the opening of the voltage-dependent Ca^2+^ channels (Raiteri and Raiteri, 2000; Nicholls, 2003). We asked whether a possible correlation exists linking the sorting of the exosomes triggered by the high-KCl stimulus to the vesicular exocytosis of transmitter(s) in depolarized synaptosomes. To answer the question, we focused on glutamate, which represents the most diffuse transmitter in the brain. Despite cortical synaptosomes being a heterogenous population, the glutamatergic ones largely prevail, so the quantification of glutamate exocytosis permits us to monitor the release efficiency in the largest subpopulation of cortical synaptosomes (Grilli et al., 2004). As expected, we detected a significant increase in the glutamate overflow in the supernatants of the depolarized cortical synaptosomes from which the exosomes were isolated. Interestingly, both the glutamate availability and the exosomal marker content was largely reduced when the external Ca^2+^ ions were omitted to levels comparable to those detected under basal, non-depolarizing, condition.

The huge correlation in terms of Ca^2+^ dependency between glutamate exocytosis and exosome release seems best interpreted by assuming that both events might involve common triggering pathway(s). To address this more directly, we focused on presynaptic receptors, which represent one of the main mechanisms of control of vesicular exocytosis (Raiteri, 2001; Langer, 2008; Pittaluga, 2019) and asked whether release-regulating receptors could also control the sorting of exosomes elicited by high KCl. We focused on presynaptic GABA_B_ receptors because of their wide distribution in cortical nerve endings (they exist on cortical GABAergic, glutamatergic, peptidergic, and dopaminergic terminals; Bonanno and Raiteri, 1993) and their ability to modulate transmitter exocytosis through the inhibition of the voltage-dependent Ca^2+^ channels. The activation with (±)-baclofen of presynaptic GABA_B_ heteroreceptors efficiently inhibited the glutamate overflow in cortical synaptosomes, as already reported in the literature (Perkinton and Sihra, 1998). The agonist, however, failed to affect the secretion of exosomes, as suggested by the finding that the immunoreactivities of the exosomal markers isolated from the supernatant of depolarized synaptosomes exposed to (±)-baclofen were superimposable to those detected in the absence of the agonist. The more likely conclusion is that the depolarization-evoked, Ca^2+^-dependent sorting of exosomes from isolated nerve endings escapes the presynaptic control of the GABA_B_ presynaptic receptors. This would imply that Ca^2+^ enters synaptosomes through accesses that are differently sensitive to the GABA_B_ receptors. Although further investigation is needed to correctly address the question, the hypothesis is supported by data in the literature showing that the N and the P/Q-type Ca^2+^ channels, which assure the influx of the divalent cation in nerve endings, are differently sensitive to the presynaptic inhibitory actions of the GABA_B_ receptors (Langer, 2008). These data unveil a multiplicity of calcium-dependent events that can occur in synaptosomes and could be differently modulated but could also lead to different functional outcomes.

To conclude, the results described in this study provide evidence supporting the release of exosomes from presynaptic structures. The structural and biochemical analysis confirmed the exosomal nature of the EVs in the synaptosomal supernatants, also unveiling their increased availability upon exposure of synaptosomes to a depolarizing stimulus. The exosomal particles show a proteomic profile distinct from that of the synaptosomes, suggesting that these nanosized vesicles differ in nature from the synaptosomes they are released from. Finally, the release of the exosomes from the synaptosomes is a Ca^2+^-dependent event, which possibly depends on the firing of the nerve terminals. Interestingly, the exosomal sorting was not controlled by presynaptic GABA_B_ receptors, suggesting that the vesicular transmitter release and the exosome outflow are independent mechanisms which develop concomitantly at nerve endings. The finding, however, would not exclude the existence of presynaptic mechanism(s) of control of the exosome sorting that would represent rather attractive target(s) for therapeutic purposes.

Exosomes have emerged in recent years as a cargo of proteins/lipids/genetic material rapidly transferred to other cells with an efficiency that would strictly depend on the kinetics of release and diffusion in the biophase. They were proposed to carry components that could be either deleterious to the course of neurological diseases (e.g., Alzheimer’s disease, Goetzl et al., 2018; Parkinson’s disease, McCormack et al., 2019; and multiple sclerosis, Sáenz-Cuesta et al., 2014) or beneficial because of their anti-inflammatory/immunomodulatory properties (Bahrini et al., 2015; Cui et al., 2018). In both cases, the possibility to pharmacologically tune the efficiency of their release and diffusion (and as a consequence, of the materials they vehiculate) by means of ligands acting at release-regulating receptors would give new therapeutic opportunities to manage the course of central disorders. Therefore, despite the disappointing results obtained with (±)-baclofen, we believe that the study of the mechanism of sorting of exosomes from nerve endings improves our knowledge on the mechanism of synaptic transmission, paving the road to new therapeutic treatments.

## Data Availability

The raw data supporting the conclusions of this article will be made available by the authors, without undue reservation.

## Abbreviations

ESCRT-I, endosomal sorting complex required for transport I; EVs, extracellular vesicles; LDH, lactate dehydrogenase; MVB, multivesicular body; PSD95, postsynaptic density protein 95; t-TBS, tris-buffered saline-tween; TEM, transmission electron microscopy; TSG101, tumor susceptibility gene 101 protein; WG, wingless.
